# Bone Metastases Detection in Patients with Breast Cancer: Does Bone Scintigraphy Add Information to PET/CT?

**DOI:** 10.1093/oncolo/oyad087

**Published:** 2023-04-08

**Authors:** Joana Cristo Santos, Miguel Henriques Abreu, Miriam Seoane Santos, Hugo Duarte, Tiago Alpoim, Inês Próspero, Susana Sousa, Pedro Henriques Abreu

**Affiliations:** University of Coimbra, CISUC, Department of Informatics Engineering, Coimbra 3030-290, Portugal; Department of Medical Oncology, Portuguese Institute of Oncology of Porto Francisco Gentil, EPE, Porto, Portugal; University of Coimbra, CISUC, Department of Informatics Engineering, Coimbra 3030-290, Portugal; Department of Nuclear Medicine, Portuguese Institute of Oncology of Porto Francisco Gentil, EPE, Porto, Portugal; Department of Medical Oncology, Portuguese Institute of Oncology of Porto Francisco Gentil, EPE, Porto, Portugal; Department of Nuclear Medicine, Portuguese Institute of Oncology of Porto Francisco Gentil, EPE, Porto, Portugal; Department of Medical Oncology, Portuguese Institute of Oncology of Porto Francisco Gentil, EPE, Porto, Portugal; University of Coimbra, CISUC, Department of Informatics Engineering, Coimbra 3030-290, Portugal

## Abstract

**Background:**

Positron emission tomography/computed tomography (PET/CT) has become in recent years a tool for breast cancer (BC) staging. However, its accuracy to detect bone metastases is classically considered inferior to bone scintigraphy (BS). The purpose of this work is to compare the effectiveness of bone metastases detection between PET/CT and BS.

**Materials and Methods:**

Prospective study of 410 female patients treated in a Comprehensive Cancer Center between 2014 and 2020 that performed PET/CT and BS for staging purposes. The image analysis was performed by 2 senior nuclear medicine physicians. The comparison was performed based on accuracy, sensitivity, and specificity on a patient and anatomical region level and was assessed using McNemar’s Test. An average ROC was calculated for the anatomical region analysis.

**Results:**

PET/CT presented higher values of accuracy and sensitivity (98.0% and 93.83%), surpassing BS (95.61% and 81.48%) in detecting bone disease. There was a significant difference in favor of PET/CT (sensitivity 93.83% vs. 81.48%), however, there is no significant difference in eliminating false positives (specificity 99.09% vs. 99.09%). PET/CT presented the highest accuracy and sensitivity values for most of the bone segments, only surpassed by BS for the cranium. There was a significant difference in favor of PET/CT in the upper limb, spine, thorax (sternum) and lower limb (pelvis and sacrum), and in favor of BS in the cranium. The ROC showed that PET/CT has a higher sensitivity and consistency across the bone segments.

**Conclusion:**

With the correct imaging protocol, PET/CT does not require BS for patients with BC staging.

## Teaser

This article compares the effectiveness of the PET/CT scan and bone scintigraphy for the detection of bone metastases in patients with breast cancer.

**Implications for Practice**

Positron emission tomography/computed tomography (PET/CT) has become in recent years an important tool for breast cancer (BC) staging. However, its accuracy to detect bone metastases is classically considered inferior to bone scintigraphy (BS).

In this article, we compare in the same patient, the effectiveness of bone metastasis detection of PET/CT and BS in patients with BC, demonstrating that we can avoid BS in BC staging when we use PET/CT.

## Introduction

Breast cancer (BC) is the most commonly diagnosed cancer and the leading cause of cancer death among women.^1,2^ Distant metastases represent the main cause of death and are common in advanced stages of the disease.^3^

Bone is the main site of metastasis in patients with BC, accounting for 20% of the distant metastasis.^4^ Bone metastases are classified as osteolytic, osteoblastic, or mixed, and are associated with considerable morbidity including pain, impaired mobility, hypercalcemia, pathological fracture, and bone marrow infiltration.^5,6^ Therefore, early detection of skeletal metastasis is essential for the management of the disease and to define staging and optimal treatment.^7^

Imaging plays a key role in the diagnosis of bone metastasis in BC, in which bone scintigraphy (BS) remains the most used modality.^8^ This conventional imaging method provides information on osteoblastic activity and skeletal vascularity, and presents preferential uptake of tracer at sites of active bone formation.^9^ Even though this technique remains popular among clinicians, it still has some limitations, namely low specificity.^10^

Positron emission tomography/computed tomography (PET/CT) has been shown to obtain improved sensitivity and specificity when compared to conventional imaging modalities.^4^ It detects the presence of cancer cells directly by quantifying metabolic activity, which allows the analysis of active tumor tissue in the whole body.^9^

BS in combination with CT is considered the gold standard for BC staging. However, the use and relevance of PET/CT in this context have increased in recent years.^11^ Even though BS and PET/CT have been applied to the detection of bone metastasis, no consensus has been established on the most suitable imaging modality for this purpose. International Guidelines recommend PET/CT for staging in patients with locally advanced disease and inflammatory carcinomas^12,13^ and the National Comprehensive Cancer Network (NCCN) guidelines suggest that BS might be omitted in certain cases when PET/CT documents bone metastases.^12^

In this article, a prospective analysis is performed to compare the effectiveness of bone metastasis detection of PET/CT and BS in patients with BC, aiming to understand the cases where the latter can be avoided.

## Materials and Methods

### Patient Selection

Prospective inclusion of patients with BC treated in a Comprehensive Cancer Center (Portuguese Institute of Oncology of Porto Francisco Gentil, EPE, Porto, Portugal) between 2014 and 2020 that performed PET/CT and BS for staging purposes. Only patients that performed the 2 exams with an interval less than 2 months—16 ± 14 days (average ± SD); range 0-60 days—were considered.^14^ This study was approved by the local Ethics and Data Protection Committees.

### Image Acquisition Protocols

#### Bone Scintigraphy

Patients were injected intravenously with 740-925 MBq of 99mTc-hydroxymethane diphosphonate (99mTc-HDP).

Whole-body planar images were acquired 2 h after injection in a 256 × 256 matrix, with a 20% window centered around the 140-keV photopeak, using a low-energy high-resolution, or general purpose parallel collimator. Additional segmental images were acquired according to the expertise and criteria defined by the attending physician.

#### PET/CT

Patients were instructed to fast for at least 6 h and to abstain from food with a high composition of sugar or carbohydrates for at least 24 h prior to the PET/CT scan. Images were acquired from the base of the skull to the mid thighs, 60 ± 10 minutes after intravenous administration of ­2-[F-18]-fluor-2-desoxi-D-glucose (18F-FDG) (3.5 a 7 MBq/kg). Blood glucose levels were <130 mg/dL, or up to a maximum of 200 mg/dL in patients with diabetes. Patients rested in a low light, warm, and quiet room in the time between the administration of the radiopharmaceuticals and the image acquisition. PET/CT scans were performed using either a Siemens Biograph 6 or a Siemens Biograph 20mCT dedicated scanner. PET scans were executed in 3D mode, after a low-dose CT scan at free breathing for attenuation and scatter correction.

### Image Analysis

The BS and PET/CT images were routinely evaluated by 2 senior nuclear medicine physicians. Consistent readings between BS and PET/CT were considered true positives. In cases where there was a discrepancy between the 2 modalities, a re-analysis of the images by other 2 independent nuclear medicine physicians was performed to validate the findings.

### Data Analysis

Clinical and pathological information about the patients and the disease were collected from electronic files by a medical oncologist.

For evaluation purposes, the comparison of the ability of BS and PET/CT to identify bone disease was performed based on accuracy, sensitivity, and specificity, at a patient and anatomical region level. In the anatomical region-based analysis, the skeleton was divided into 5 regions composed of 10 bone segments: cranium, upper limb (scapulae, clavicle, and humerus), spine, thorax (rib cage and sternum), and lower limb (pelvis and sacrum, femur, tibia, and fibula).

The bone metastases distribution was categorized depending on the number of bone lesions. Accordingly, 3 categories were considered: “isolated metastasis” (only one lesion), “less than 5 bone lesions” (between 2 and 5 lesions), and “more than 5 lesions.”

### Statistical Analysis

To compare the effectiveness (accuracy, sensitivity, and specificity) of bone metastasis detection of PET/CT and BS, the McNemar’s Test was used.^15,16^ A *P*-value < .05 was considered statistically significant.

An average receiver operating characteristic curve (ROC) curve and the corresponding area under the curve (AUC) were calculated for the anatomical region analysis. The ROC curve of each bone segment was combined to obtain a single ROC curve to study the bone disease detection.

## Results

The majority of the 410 female patients included in the study presented ductal carcinomas (326/410, 79.5%), grade 3 (283/410, 69.0%), T2 (193/410, 47.1%), N1 (151/410, 36.8%), and M0 (298/410, 72.7%). Patients also presented ER and PR expression (300/410, 73.2% and 246/410, 60.0%, respectively), whereas the co- expression of HER2 was not observed (302/410, 73.7%). The clinical and pathological characteristics of the patients are described in Table 1.

**Table 1.** Patient and disease characteristics (N = 410).

Characteristics	n (%)
Age, years	
Mean	54 ± 13
Range	25-85
Histology	
Ductal carcinoma	326 (79.5)
Lobular carcinoma	44 (10.8)
Mixed	5 (1.2)
Other	35 (8.5)
Grade	
G1	13 (3.2)
G2	114 (27.8)
G3	283 (69.0)
TNM Staging^a^	
T	
T1	77 (18.8)
T2	193 (47.1)
T3	93 (22.7)
T4	47 (11.4)
N	
N0	99 (24.1)
N1	151 (36.8)
N2	104 (25.4)
N3	56 (13.7)
M	
M0	298 (72.7)
M1	112 (27.3)
Stage	
I	21 (5.1)
II	142 (34.7)
III	135 (32.9)
IV	112 (27.3)
ER status^b^	
Positive	300 (73.2)
Negative	110 (26.8)
PR status^c^	
Positive	246 (60.0)
Negative	164 (40.0)
HER2 status	
Positive	108 (26.3)
Negative	302 (73.7)

^a^According to AJCC 8th edition^17^

Abbreviations: ER, estrogen receptor; PR, progesterone receptor.

Distant metastases were identified in 112/410 (27.3%) patients. Bone metastasis was observed in 81/112 patients (72.3%) and was the only distant metastasis site for 40/81 patients, representing 49.8% of all the patients with bone disease.

### Patient-Based Analysis

The results of the patient-based analysis are described in Table 2. In total, PET/CT misdiagnosed 5/81 patients (6.2%) and BS misdiagnosed 15/81 patients (18.5%). In the case of PET/CT, 1 patient was misdiagnosed due to the ­non-detection of a cranium metastasis, whereas the other 4 patients presented bone lesions that did not show metabolic activity (whose presence was confirmed in BS). In the case of BS, the exam was not capable of diagnosing an isolated metastasis in 7 patients, was not capable of identifying various metastases in 4 patients, and was not capable of identifying diffuse bone disease in 4 patients. Consequently, PET/CT obtained an accuracy of 98.05%, surpassing the performance of BS (95.61%).

**Table 2.** Patient-based analysis.

	PET/CT (%)	BS (%)	*P*-value
Accuracy	98.05	95.61	.0775
Sensitivity	93.83	81.48	**.0442**
Specificity	99.09	99.09	.6831

The statistically significant *P*-values are in bold.

There is a significant difference in favor of PET/CT in bone metastases detection (sensitivity 93.83% vs. 81.48%, *P* = .0442). However, there is no significant difference in eliminating false positives (specificity 99.09% vs. 99.09%, *P* = .6831).

### Anatomical Region-Based Analysis

The bone metastasis distribution according to the pre-defined categories was as follows: 16 patients with isolated metastasis (19.8%), 26 patients with less than 5 lesions (32.1%), and 39 patients with more than 5 lesions (48.1%).

The distribution of metastases by anatomical region and segment and the results of region-based analysis are presented in Tables 3 and 4.

**Table 3.** Metastasis by anatomical region and by segment.

Anatomical region	n (%)
Cranium	23 (6.1)
Upper limb	
Scapulae	33 (8.8)
Clavicle	24 (6.4)
Humerus	31 (8.2)
Spine	68 (18.1)
Thorax	
Rib cage	55 (14.6)
Sternum	38 (10.1)
Lower limb	
Pelvis and sacrum	64 (17.0)
Femur	37 (9.9)
Tibia and fibula	3 (0.8)
Total	376 (100)

**Table 4.** Anatomical region-based analysis.

		True positives	Accuracy	*P*-value	Sensitivity	*P*-value
Cranium	PET/CT	9/23	82.72	**.0153**	39.13	**.0153**
	BS	21/23	96.30		91.30	
Upper limb						
Scapulae	PET/CT	33/33	100.00	**.0001**	100.00	**.0001**
	BS	11/33	72.84		33.33	
Clavicle	PET/CT	24/24	100.00	**.0001**	100.00	**.0001**
	BS	6/24	77.78		25.00	
Humerus	PET/CT	29/31	97.53	**.0059**	93.55	**.0059**
	BS	17/31	82.72		54.84	
Spine	PET/CT	66/68	97.53	**.0001**	97.06	**.0001**
	BS	46/68	72.84		67.65	
Thorax						
Rib Cage	PET/CT	48/55	91.36	.0550	87.27	.0550
	BS	38/55	79.01		69.09	
Sternum	PET/CT	36/38	96.29	**.0008**	94.74	**.0008**
	BS	20/38	76.53		52.63	
Lower limb						
Pelvis and Sacrum	PET/CT	61/64	96.29	**.0001**	95.31	**.0001**
	BS	40/64	70.37		62.50	
Femur	PET/CT	33/37	95.06	.0523	89.19	.0523
	BS	24/37	83.95		64.86	
Tibia and Fibula	PET/CT	1/3	97.53	1.000	33.33	1.000
	BS	3/3	98.76		66.66	

The statistically significant *P*-values are in bold.

The bone segments that presented the highest metastasis incidence rates were the spine (68/376, 18.1%), the lower limb (pelvis and sacrum) (64/376, 17.0%), and the thorax (rib cage) (55/376, 14.6%). The lowest metastasis incidence rates were obtained for the lower limb (tibia and fibula) (3/376, 0.8%).

PET/CT presented the highest accuracy and sensitivity values for most of the bone segments, only surpassed by BS for the cranium.

On the one hand, there is a significant difference in favor of PET/CT in bone metastasis detection in the upper limb (scapulae, clavicle, and humerus), spine, thorax (sternum), and lower limb (pelvis and sacrum). On the other hand, there is a significant difference in favor of BS in bone metastasis detection of the cranium. Detection was similar in the thorax (rib cage), lower limb (femur), and lower limb (tibia and fibula).

**Figure 1.**
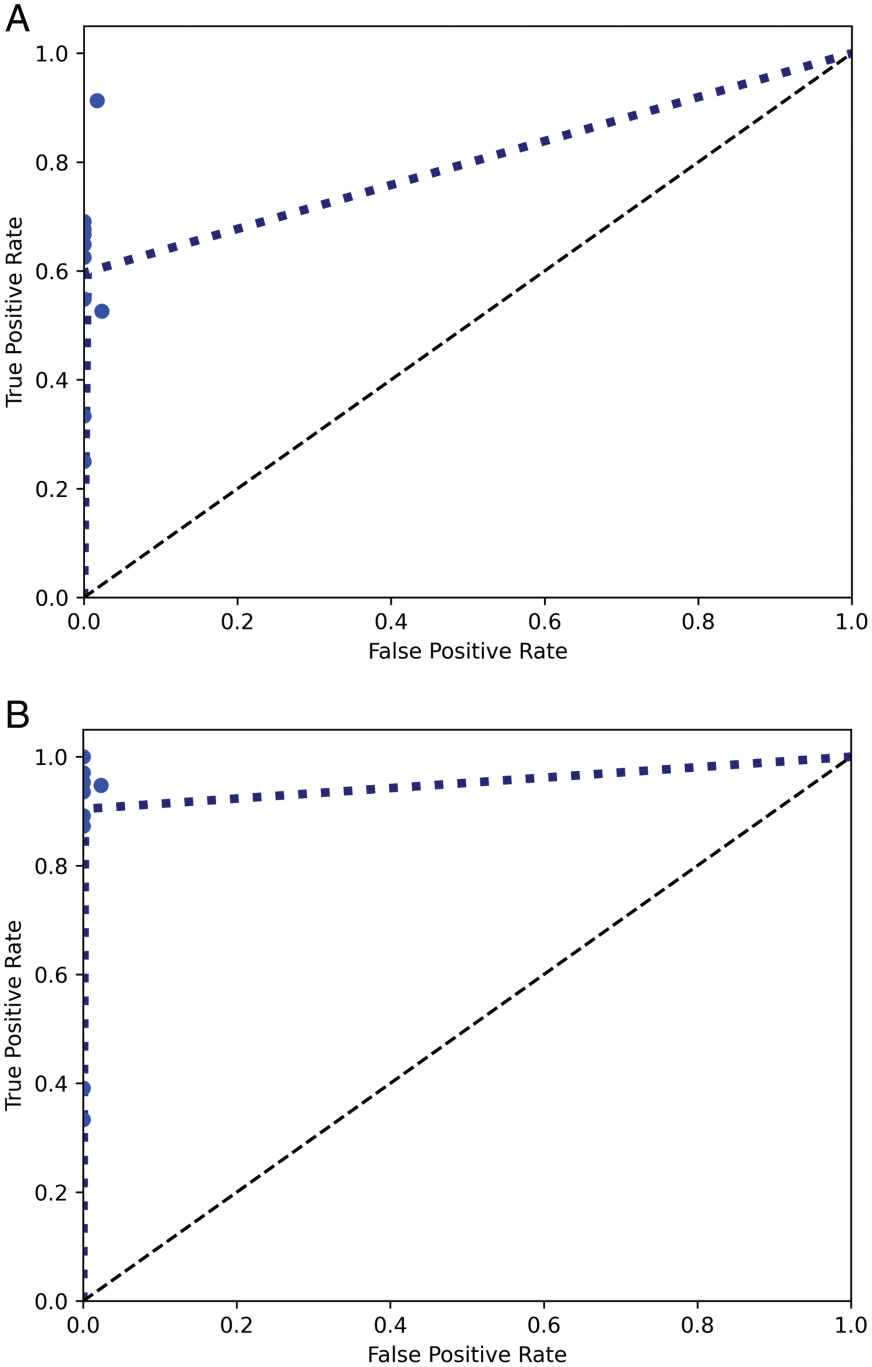
Average ROC curves. The dots represent the values for each bone segment and the line corresponds to the average ROC curve. (**a**) ROC curve for PET/CT (AUC = 0.9509). (**b**) ROC curve for BS (AUC = 0.7969).

The ROC curves (Fig. 1) show that PET/CT presents higher sensitivity results (ie, higher true positive rates), and a higher consistency across the different bone segments. BS, on the other hand, presents lower values of sensitivity and no consistency throughout the different bone segments, presenting sensitivity values between 0.2 and 1.

## Discussion

Staging of early patients with BC with increased risk for metastases is essential to define prognosis and establish the best therapeutical approach. International guidelines advocate performing thoracoabdominal CT as well as BS as part of initial BC staging.^12,13,18^ Since bone is the main local of metastases, present in almost 50%-70% of the patients with advanced disease,^19,20^ procedures must have a high sensitivity to identify this pathology since its early detection could also help to minimize skeletal-related effects that decrease patients’ quality of life.^21^

For many years, it has been established that osteolytic lesions are not identified in BS unless the bone marrow already has a huge destruction.^22^ As a result, hybrid imaging techniques that associate anatomical and metabolic imaging (eg, PET/CT) could be beneficial.^23-25^

Our study raises the question of whether BS is still necessary for BC bone staging when PET/CT is available. To our knowledge, this is the biggest prospective study that addresses this question, stratifying the analysis by patient and anatomical region.

Even though PET/CT presents the best overall effect in the bone metastases detection, the low performance in the detection of cranium lesions requires further analysis. This issue may be caused by the imaging protocol of PET/CT, which does not require the imaging of these segments. Accordingly, this modification of the image acquisition protocol could be enough to solve this handicap.

In a meta-analysis with 13 articles,^21^ PET/CT seems equal to BS for diagnosis of bone metastases in patients with BC, considering a per-patient basis. However, on a per-lesion basis, PET/CT had lower sensitivity and higher specificity than BS. In this analysis, the authors stated that due to limitations such as the small number of datasets available for PET/CT, and the small sample size of included studies, it was not possible to devise standard recommendations for clinical practice or future research. Particularly in patients with BC with bone disease, PET/CT performed at baseline (staging), could also be important to predict overall survival, being able to define a subgroup of patients that will live longer.^24^

In a small survey of 7 patients with BC, where 41 bones were analyzed,^26^ BS has shown limited sensitivity in the detection of metastases (between 28.6% and 36.6%). Although PET/CT was not used, positron emission ­tomography-magnetic resonance imaging (PET/MRI) and magnetic resonance imaging (MRI) have obtained ­high-sensitivity results (100%).

The comparison of PET/CT and MRI has conflicting results,^27-29^ with both techniques generally showing a good performance. PET/MRI is not transversely available, but the idea to add functional information is very attractive, and is being tested in 2 clinical trials to support BC staging.^30^

## Conclusion

PET/CT surpasses BS in the detection of BC bone metastases and must be the technique of choice to stage these patients.

## Data Availability

The data underlying this article cannot be shared publicly due to the privacy of individuals that participated in the study.
